# Systemic sclerosis and risk of bronchiectasis: a nationwide longitudinal cohort study

**DOI:** 10.1186/s13075-023-03189-2

**Published:** 2023-10-23

**Authors:** Bumhee Yang, Bo-Guen Kim, Kyungdo Han, Jin-Hyung Jung, Ji Hyoun Kim, Dong Won Park, Sang-Heon Kim, Eung-Gook Kim, Jang Won Sohn, Ho Joo Yoon, Hayoung Choi, Hyun Lee

**Affiliations:** 1 Division of Pulmonary and Critical Care Medicine, Department of Internal Medicine, Chungbuk National University Hospital, Chungbuk National University College of Medicine, Cheongju, Republic of Korea; 2 Division of Pulmonary Medicine and Allergy, Department of Internal Medicine, Hanyang University College of Medicine, 222-1, Wangsimni-ro, Seoul, Seongdong-gu 04763 Republic of Korea; 3 Department of Statistics and Actuarial Science, Soongsil University, Seoul, Republic of Korea; 4 Department of Biostatistics, College of Medicine, Catholic University of Korea, Seoul, Republic of Korea; 5 Division of Rheumatology, Department of Internal Medicine, Chungbuk National University Hospital, Cheongju, Republic of Korea; 6 Department of Biochemistry, Chungbuk National University College of Medicine, Cheongju, Republic of Korea; 7 Division of Pulmonary, Allergy, and Critical Care Medicine, Department of Internal Medicine, Hallym University Kangnam Sacred Heart Hospital, Hallym University College of Medicine, 1 Singil-ro, Seoul, Yeongdeungpo-gu 07441 Republic of Korea

**Keywords:** Systemic sclerosis, Bronchiectasis, Epidemiology, Risk

## Abstract

**Background:**

The association between systemic sclerosis and the development of bronchiectasis is unclear. This study aimed to compare the risk of bronchiectasis between individuals with systemic sclerosis and those without using a nationwide longitudinal dataset.

**Methods:**

Using the Korean National Health Insurance Service dataset between 2010 and 2017, we identified 4845 individuals aged ≥ 20 years with systemic sclerosis and 24,225 without systemic sclerosis who were matched 1:5 by age and sex. They were followed up until the date of a bronchiectasis diagnosis, death, or December 31, 2019, whichever came first.

**Results:**

During a median follow-up period of 6.0 (interquartile range, 3.2–8.7) years, 5.3% of the systemic sclerosis cohort and 1.9% of the matched cohort developed bronchiectasis, with incidence rates of 9.99 and 3.23 per 1000 person-years, respectively. Even after adjusting for potential confounders, the risk of incident bronchiectasis was significantly higher in the systemic sclerosis cohort than in the matched cohort (adjusted hazard ratio 2.63, 95% confidence interval 2.22–3.12). A subgroup analysis of individuals with systemic sclerosis revealed that the risk of incident bronchiectasis was notably higher in younger individuals aged 20–39 years (*P* for interaction = 0.048) and in those without other coexisting connective tissue diseases (*P* for interaction = 0.006) than in their counterparts.

**Conclusions:**

The risk of incident bronchiectasis is higher in individuals with systemic sclerosis than those without. Bronchiectasis should be considered one of the pulmonary manifestations related to systemic sclerosis.

**Supplementary Information:**

The online version contains supplementary material available at 10.1186/s13075-023-03189-2.

## Background

Systemic sclerosis is an autoimmune condition characterized by cutaneous fibrosis and multi-organ involvement, resulting in significant morbidity and mortality [1]. Before the availability of angiotensin-converting enzyme inhibitor therapeutics, renal disease was the most common cause of death [2]. However, pulmonary complications such as pulmonary arterial hypertension and interstitial lung disease (ILD) [3, 4], are the leading causes of death in patients with systemic sclerosis in recent years, suggesting the importance of managing comorbid pulmonary conditions [5–7].

Non-cystic fibrosis bronchiectasis (hereafter referred to as bronchiectasis) is a chronic lung disease characterized radiologically by permanent bronchial dilatation and clinically by the presence of cough, sputum, and recurrent chest infections [8]. A few systemic sclerosis cohort studies have reported that bronchiectasis is a common pulmonary comorbidity in patients with systemic sclerosis, ranging from 3.3 to 59.1% [9–11]. However, it is not well known whether the risk of incident bronchiectasis is higher in patients with systemic sclerosis due to small study populations and the absence of comparable controls [9–11]. Furthermore, bronchiectasis itself is known to be associated with increased mortality [12], and an inflammatory process in the lungs could provoke autoimmune responses in rheumatoid arthritis potentially creating a worse prognosis [13, 14]. This suggests that bronchiectasis may affect the prognosis of systemic sclerosis. In this regard, we need to consider comorbid bronchiectasis in patients with systemic sclerosis and an appropriate starting point would be to determine whether individuals with systemic sclerosis are at a higher risk of bronchiectasis than those without systemic sclerosis.

Therefore, this study aimed to compare the risk of incident bronchiectasis between a systemic sclerosis cohort and an age and sex-matched non-systemic sclerosis cohort drawn from a large nationally representative longitudinal database in Korea.

## Materials and methods

### Data source

This study used a dataset provided by the Korean National Health Insurance Service (NHIS), a universal insurance provider managed by the government that covers 97% of the Korean population, approximately 50 million people. The NHIS dataset includes information on socioeconomics, demographic variables, healthcare utilization, health examination findings, disease diagnosis based on the 10th revision of the International Classification of Disease (ICD-10) codes, and medical treatment and procedures [15]. The NHIS database includes a variety of medical and health information and has been widely used in various epidemiologic studies to identify risk factors for diseases [16, 17].

Our study protocol was approved by the Institutional Review Board of Chungbuk National University Hospital (No. 2023-01-014). The requirement for informed consent was waived because the NHIS database uses an anonymous patient identification system.

### Study population and characteristics

**Fig. 1:**
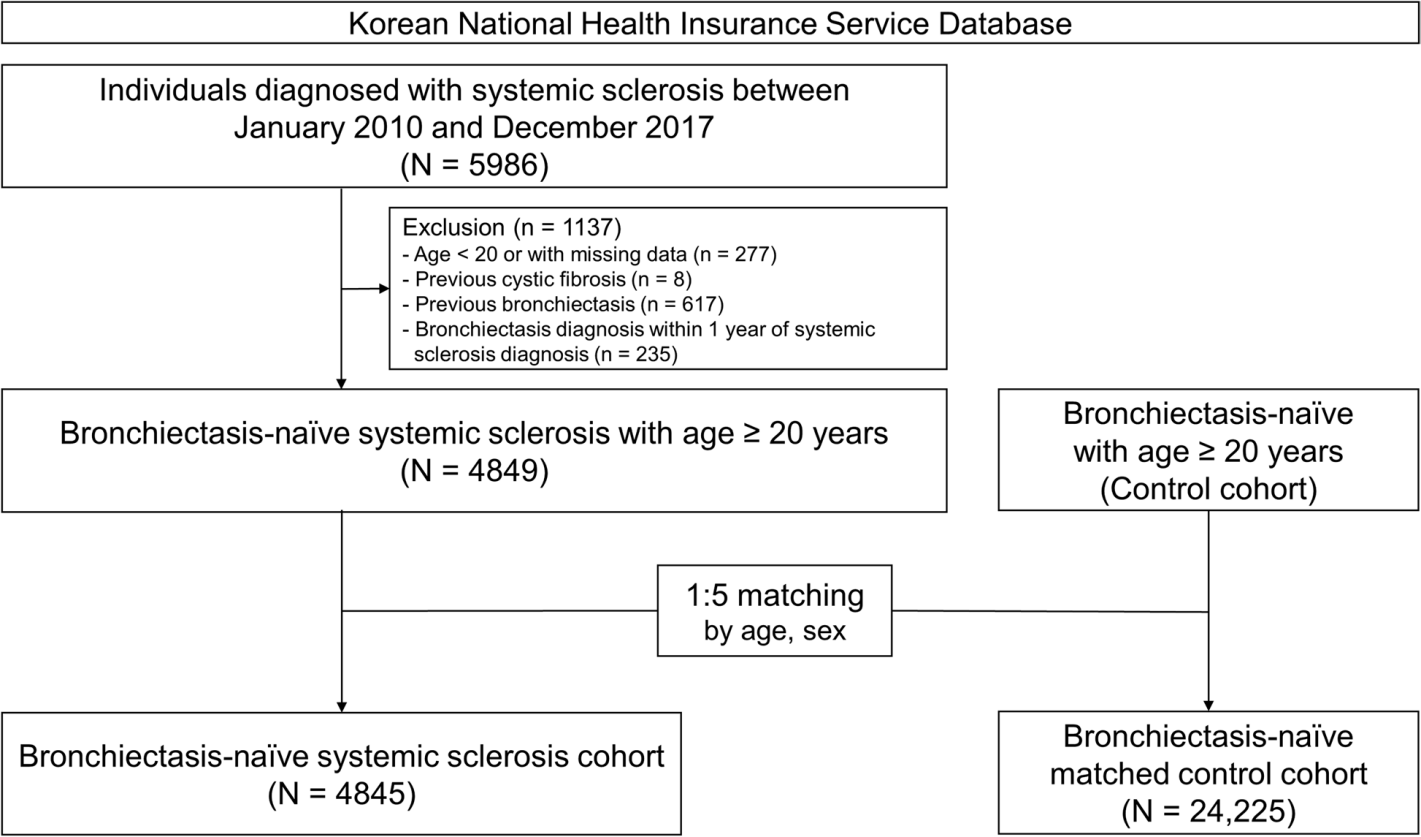
Flowchart for the study

We initially included 5986 individuals who were diagnosed with systemic sclerosis between 2010 and 2017. After excluding those with missing data or who were younger than 20 years (*n* = 277), those who had previously been diagnosed with cystic fibrosis (*n* = 8), those who had previously been diagnosed with bronchiectasis (*n* = 617), and those who were diagnosed with bronchiectasis within 1 year after systemic sclerosis diagnosis (*n* = 235), we enrolled 4849 for the systemic sclerosis cohort. Of these 4849 individuals, 4845 were eligible for 1:5 age and sex matching. Thus, the study enrolled 4845 individuals for the systemic sclerosis cohort and matched these by age and sex to 24,225 control subjects who had not been diagnosed with systemic sclerosis or bronchiectasis (Fig. 1). The control group had health insurance claims for diseases other than systemic sclerosis in the same year that the matched participants with systemic sclerosis were diagnosed with the disease. Thus, the date of healthcare utilization in the matched year was regarded as the index date for the control group. Additionally, participants who had been diagnosed with cystic fibrosis and those who developed bronchiectasis within one year of enrollment were excluded from the control group.

### Study exposure

The exposure of this study was systemic sclerosis, of which the definition required (1) at least one hospital visit with the ICD-10 diagnostic code M34.0, and 2) registration with the Rare and Intractable Disease program (code V138) [17]. Since January 2006, patients with rare and intractable diseases (e.g., malignancy, tuberculosis, and connective tissue disease [CTD]) have been registered in the Individual Copayment Beneficiaries Program in the Republic of Korea to reduce the burden of medical expenses [16, 18]. Thus, the validity of the systemic sclerosis diagnosis was strictly reviewed by the Health Insurance Review and Assessment Service. The date of registration in the Rare and Intractable Disease program for systemic sclerosis was defined as the index date.

### Study outcome

The outcome of this study was the incidence of bronchiectasis. Bronchiectasis was defined as claims under the ICD-10 diagnosis code J47 without a concomitant diagnosis of cystic fibrosis (ICD-10 code E84) in any form of healthcare utilizations (i.e., outpatient department visits, emergency room visits, and hospitalization) [16, 19–21]. The study participants were followed up from the index date to the date of bronchiectasis incidence, censored date, or December 31, 2019 (end date of the study).

### Covariates

The definitions of comorbidities (diabetes mellitus, hypertension, dyslipidemia, end-stage renal disease, ischemic heart disease, congestive heart failure, asthma, and chronic obstructive pulmonary disease [COPD]) were based on ICD-10 codes as previously described [19, 20, 22–24]. Additionally, tuberculosis and other CTDs (rheumatoid arthritis, systemic lupus erythematosus, dermatomyositis, mixed CTD, and polymyalgia rheumatica) were defined using ICD-10 codes as well as registration with the national rare intractable disease supporting program [17, 23, 24]. Household income was categorized into quartiles based on insurance premium levels (in Korea, insurance premiums are determined by income level), with those covered by Medical Aid (poorest 3%) being merged into the lowest income quartile [23–25].

### Statistical analysis

Descriptive statistics are presented as numbers (percentages) for categorical variables and mean ± standard deviations (SDs) for continuous variables. We compared the two groups using the *χ*^2^ test for categorical variables, and *t*-test for continuous variables. The incidence rates of bronchiectasis were calculated by dividing the number of incident events by the total follow-up period (1000 person-years). A cumulative incidence plot was used to compare the incidence of bronchiectasis between the systemic sclerosis and matched cohorts, and a log-rank test was used to evaluate significant differences between the groups.

The risk of incident bronchiectasis in the systemic sclerosis cohort compared to the matched cohort was estimated using univariable and multivariable Cox proportional hazards regression analyses. Model 1 was an unadjusted model. Model 2 was adjusted for sex, age, income, diabetes mellitus, hypertension, and dyslipidemia. Model 3 was additionally adjusted for end-stage renal disease, ischemic heart disease, congestive heart failure, COPD, asthma, tuberculosis, and other CTDs. Stratified analyses were performed by sex, age, income, and comorbidities (COPD, asthma, tuberculosis, and other CTDs). Additionally, to exclude traction bronchiectasis associated with ILD, we performed a sensitivity analysis to assess the risk of bronchiectasis without a diagnostic code for ILD (J84). A two-sided *P* value < 0.05 was considered statistically significant. All statistical analyses were performed using SAS version 9.4 (SAS Institute Inc., Cary, NC, USA).

## Results

### Baseline characteristics

The mean age of the study population was 53.5 years (SD, 12.6 years) and 15.2% were male. The proportions of pulmonary and extra-pulmonary comorbidities were higher in the systemic sclerosis cohort than the matched cohort (*p* < 0.001 for all), except for diabetes mellitus (*p* = 0.840). Moreover, the proportions of other CTDs (rheumatoid arthritis, systemic lupus erythematosus, dermatomyositis, and mixed CTD) were also higher in the systemic sclerosis cohort than the matched cohort (*p* < 0.001 for all), except for polymyalgia rheumatica (*p* = 0.206) (Table 1).

**Table 1:** Baseline characteristics of the study population

	Total (*N* = 29,070)	Systemic sclerosis cohort (*n* = 4845)	Matched cohort (*n* = 24,225)	*P* value
Male sex	4422 (15.2)	737 (15.2)	3685 (15.2)	> 0.999
Age, years	53.5 ± 12.6	53.4 ± 12.6	53.5 ± 12.6	> 0.999
20–39	4050 (13.9)	675 (13.9)	3375 (13.9)	
40–64	19,338 (66.5)	3223 (66.5)	16,115 (66.5)	
≥ 65	5682 (19.6)	947 (19.6)	4735 (19.6)	
Extra-pulmonary comorbidities				
Diabetes mellitus	2355 (8.1)	389 (8.0)	1966 (8.1)	0.840
Hypertension	8411 (28.9)	2562 (52.9)	5849 (24.1)	< 0.001
Dyslipidemia	4836 (16.6)	922 (19.0)	3914 (16.2)	< 0.001
ESRD	88 (0.3)	35 (0.7)	53 (0.2)	< 0.001
IHD	1991 (6.8)	720 (14.7)	1271 (5.3)	< 0.001
CHF	539 (1.8)	231 (4.8)	308 (1.3)	< 0.001
Pulmonary comorbidities				
COPD	2516 (8.6)	959 (19.8)	1557 (6.4)	< 0.001
Asthma	3341 (11.5)	901 (18.6)	2440 (10.1)	< 0.001
Previous pulmonary tuberculosis	52 (0.2)	24 (0.5)	28 (0.1)	< 0.001
CTDs other than systemic sclerosis	558 (1.9)	472 (9.7)	86 (0.4)	< 0.001
Rheumatoid arthritis	200 (0.7)	127 (2.6)	73 (0.3)	< 0.001
Systemic lupus erythematous	246 (0.8)	234 (4.8)	12 (0.1)	< 0.001
Dermatomyositis	27 (0.1)	27 (0.6)	0 (0)	< 0.001
Mixed connective tissue disease	112 (0.4)	112 (2.3)	0 (0)	< 0.001
Polymyalgia rheumatica	2 (0.01)	1 (0.02)	1 (0)	0.206

Data are presented as number (percentage) or mean ± standard deviation. CTDs other than systemic sclerosis were rheumatoid arthritis, systemic lupus erythematous, dermatomyositis, mixed connective tissue disease, or polymyalgia rheumatica

*Abbreviations*: *ESRD* End-stage renal disease, *IHD* Ischemic heart disease, *CHF* Congestive heart failure, *COPD* Chronic obstructive lung disease, *CTD* Connective tissue disease

### Incidence and risk of bronchiectasis

**Fig. 2:**
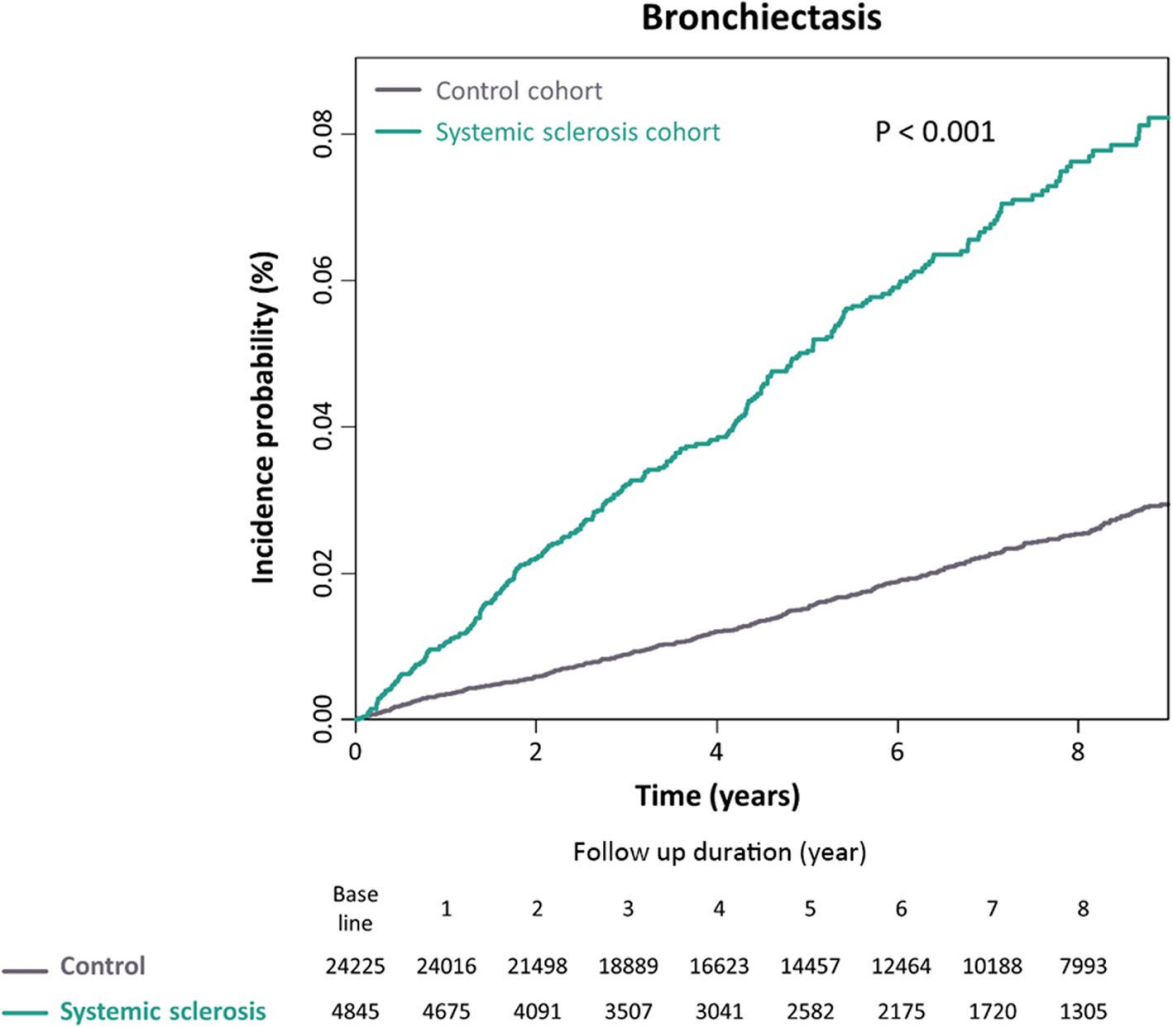
Cumulative incidence probability of bronchiectasis (/100,000 person-years) in systemic sclerosis and matched cohorts

During the median follow-up period of 6.0 (interquartile range, 3.2–8.7) years, 5.3% (*n* = 259/4845) of the systemic sclerosis cohort and 1.9% (*n* = 455/24,225) of the matched cohort developed bronchiectasis, with incidence rates of 9.99 and 3.23 per 1000 person-years, respectively (Table 2). A cumulative incidence plot depicts a significantly higher incidence of bronchiectasis in the systemic sclerosis cohort than in the matched cohort (a log-rank *p* < 0.001; Fig. 2). Even after adjusting for potential confounders, the risk of incident bronchiectasis was also significantly higher in the systemic sclerosis cohort than in the matched cohort: unadjusted hazard ratio (HR) in Model 1 3.10, 95% confidence interval [CI], 2.66–3.61; adjusted HR in Model 2 3.08, 95% CI 2.63–3.61; adjusted HR in Model 3 2.63, 95% CI 2.22–3.12) (Table 2).

**Table 2:** Risk of bronchiectasis according to the presence or absence of systemic sclerosis

	*N*	Incident bronchiectasis (*n*)	Duration (PY)	IR per 1000 PY	HR (95% CI)		
					Model 1	Model 2	Model 3
Matched cohort	24,225	455	140,863.73	3.23	1 (reference)	1 (reference)	1 (reference)
Systemic sclerosis cohort	4845	259	25,926.36	9.99	3.10 (2.66–3.61)	3.08 (2.63–3.61)	2.63 (2.22–3.12)

Data are presented as number, rate, or harzard ratio (95% confidence interval)

Model 1 was an unadjusted model; Model 2 was adjusted for sex, age, income, diabetes mellitus, hypertension, and dyslipidemia; Model 3 was additionally adjusted for ESRD, IHD, CHF, COPD, asthma, tuberculosis, and CTDs other than systemic sclerosis. CTDs other than systemic sclerosis were rheumatoid arthritis, systemic lupus erythematous, dermatomyositis, mixed connective tissue disease, or polymyalgia rheumatica

*Abbreviations*: *HR* Hazard ratio, *CI* Confidence interval, *PY* Person-years, *IR* Incidence rate, *ESRD* End-stage renal disease, *IHD* Ischemic heart disease, *CHF* Congestive heart failure, *COPD* Chronic obstructive pulmonary disease, *CTD* Connective tissue disease

### Subgroup analysis

As shown in Table 3, sex, income, and pulmonary comorbidities (COPD, asthma, or tuberculosis) did not have a significant impact on the association between systemic sclerosis and bronchiectasis development. In contrast, age and the presence of other CTDs had a significant interaction on the association of systemic sclerosis with bronchiectasis development. The risk of incident bronchiectasis was higher in younger individuals aged 20–39 years (*P* for interaction = 0.048) and in those without other coexisting CTDs (*P* for interaction = 0.006) than in their counterparts. Subgroup analysis according to other comorbidities is shown in Supplementary Table 3.

**Table 3:** Subgroup analysis of the risk of bronchiectasis

Subgroups	Systemic sclerosis	*N*	Incident bronchiectasis (*n*)	Duration (PY)	IR per 1000 PY	HR (95% CI)		
						Model 1	Model 2	Model 3
**Sex**								
Male	No	3685	67	20,814	3.22	1 (reference)	1 (reference)	1 (reference)
	Yes	737	27	3712	7.27	4.93 (3.30–7.36)	2.28 (1.46–3.58)	1.94 (1.23–3.06)
Female	No	20,540	388	120,049	3.23	Reference	1 (reference)	1 (reference)
	Yes	4108	232	22,214	10.44	2.57 (1.97–3.34)	3.21 (2.71–3.80)	2.74 (2.29–3.28)
*P* for interaction						0.140	0.163	0.155
**Age, years**								
20–39	No	3375	14	21,600	0.65	1 (reference)	1 (reference)	1 (reference)
	Yes	675	18	4131	4.36	6.73 (3.34–13.53)	6.49 (3.22–13.04)	5.64 (2.79–11.40)
40–64	No	16,115	283	93,934	3.01	1 (reference)	1 (reference)	1 (reference)
	Yes	3223	171	17,427	9.81	3.26 (2.70–3.94)	3.17 (2.60–3.86)	2.68 (2.18–3.29)
≥ 65	No	4735	158	25,329	6.24	1 (reference)	1 (reference)	1 (reference)
	Yes	947	70	4366	16.03	2.58 (1.94–3.41)	2.56 (1.93–3.40)	2.21 (1.65–2.95)
*P* for interaction						0.036	0.048	0.048
**Low income**								
Others	No	18,225	332	106,627	3.11	1 (reference)	1 (reference)	1 (reference)
	Yes	3655	194	19,744	9.83	3.15 (2.64–3.77)	3.08 (2.57–3.70)	2.63 (2.17–3.18)
Q1, low	No	6000	123	34,235	3.59	1 (reference)	1 (reference)	1 (reference)
	Yes	1190	65	6182	10.51	2.93 (2.17–3.95)	3.06 (2.25–4.15)	2.62 (1.92–3.58)
*P* for interaction						0.673	0.966	0.995
*Comorbidities*								
**COPD**								
No	No	22,668	390	132,298	2.95	1 (reference)	1 (reference)	1 (reference)
	Yes	3886	177	21,010	8.42	2.86 (2.39–3.41)	2.87 (2.39–3.45)	2.67 (2.21–3.23)
Yes	No	1557	65	8564	7.59	1 (reference)	1 (reference)	1 (reference)
	Yes	959	82	4915	16.68	2.19 (1.58–3.04)	2.57 (1.84–3.57)	2.48 (1.78–3.46)
*P* for interaction						0.163	0.555	0.694
**Asthma**								
No	No	21,785	378	127,582	2.96	1 (reference)	1 (reference)	1 (reference)
	Yes	3944	189	21,470	8.80	2.97 (2.40–3.54)	2.97 (2.48–3.56)	2.65 (2.19–3.20)
Yes	No	28	1	141	7.06	1 (reference)	1 (reference)	1 (reference)
	Yes	24	4	108	36.84	5.22 (0.58–46.75)	6.02 (0.67–53.99)	6.01 (0.66–54.04)
*P* for interaction						0.633	0.543	0.459
**Tuberculosis**								
No	No	24,197	454	140,722	3.23	1 (reference)	1 (reference)	1 (reference)
	Yes	4821	255	25,817	9.88	3.06 (2.62–3.57)	3.04 (2.59–3.57)	2.61 (2.21–3.10)
Yes	No	28	1	141	7.06	1 (reference)	1 (reference)	1 (reference)
	Yes	24	4	108	36.84	5.22 (0.58–46.75)	6.02 (0.67–53.99)	6.01 (0.66–54.04)
*P* for interaction						0.633	0.543	0.459
**CTDs other than systemic sclerosis**								
No	No	24,139	448	140,414	3.19	1 (reference)	1 (reference)	1 (reference)
	Yes	4373	231	23,482	9.84	3.08 (2.63–3.61)	3.04 (2.58–3.59)	2.71 (2.28–3.21)
Yes	No	86	7	448	15.60	1 (reference)	1 (reference)	1 (reference)
	Yes	472	28	2444	11.46	0.73 (0.32–1.68)	0.92 (0.40–2.11)	0.83 (0.36–1.90)
*P* for interaction						0.001	0.005	0.006

Data are presented as hazard ratio (95% confidence interval)

Model 1 was an unadjusted model; Model 2 was adjusted for sex, age, income, diabetes mellitus, hypertension, and dyslipidemia; and Model 3 was additionally adjusted for ESRD, IHD, CHF, COPD, asthma, tuberculosis, and CTDs other than systemic sclerosis

CTDs other than systemic sclerosis were rheumatoid arthritis, systemic lupus erythematosus, dermatomyositis, mixed connective tissue disease, or polymyalgia rheumatica

*Abbreviations*: *HR* Hazard ratio, *CI* Confidence interval, *PY* Person-years, *IR* Incidence rate, *ESRD* End-stage renal disease, *IHD* Ischemic heart disease, *CHF* Congestive heart failure, *COPD* Chronic obstructive pulmonary disease, *CTD* Connective tissue disease

## Discussion

To the best of our knowledge, this is the largest comprehensive study to evaluate the risk of bronchiectasis in systemic sclerosis by using a nationwide dataset. In this study, individuals with systemic sclerosis had a 9.99 per 1000 person-years of bronchiectasis incidence rate, which is 3-fold higher compared to that in those without systemic sclerosis (3.23 per 1000 person-years). Additionally, the risk of bronchiectasis development in systemic sclerosis was especially higher in younger individuals and in those without concomitant CTDs than in their counterparts.

Previous studies reporting the prevalence of bronchiectasis in patients with systemic sclerosis were based on small cohorts of systemic sclerosis (the largest study population was 256). In this regard, the prevalence of bronchiectasis in systemic sclerosis was reported to range widely from 3.3 to 59.1% [9–11]. To overcome the limitations of small study populations in previous studies, our study included a large number of individuals with systemic sclerosis (*n* = 4485) and revealed that about 5.5% of individuals with systemic sclerosis developed new bronchiectasis during a median 6-year follow-up period. Furthermore, by comparing the incidence of bronchiectasis between systemic sclerosis and control groups, we could reveal that systemic sclerosis could be an important etiology of bronchiectasis.

Several potential hypotheses that can explain the development of bronchiectasis in patients with systemic sclerosis exist. First, approximately 90% of patients with systemic sclerosis have gastrointestinal tract involvement [26], and the most common site is the esophagus (80%) [27]. Esophageal dysmotility could be associated with the aspiration of upper gastrointestinal contents into the airways [28]. Thus, aspiration of gastrointestinal contents might result in local bronchial inflammation, immune cell recruitment, and free radical release, which may be followed by bronchial wall damage and a consequent irreversible abnormal bronchial wall dilatation, a characteristic of bronchiectasis [29]. Second, in other CTDs, such as rheumatoid arthritis, previous studies have suggested that precedent systemic inflammation leads to the development of bronchiectasis. This notion is supported by the association between antibodies against citrullinated protein antigens and a higher risk of bronchiectasis [30] or between longer rheumatoid arthritis duration and bronchiectasis [31, 32]. This phenomenon may also explain the association between systemic sclerosis and the development of bronchiectasis. Third, the use of immunosuppressants in systemic sclerosis might be related to recurrent respiratory infection, which is an important cause of bronchiectasis [33]. Fourth, a mechanism by which sclerosis occurs within the bronchial wall, impairing ciliary contractility, and pathogen clearance, is also proposed as a hypothesis [34].

Interestingly, our subgroup analysis showed that the association between systemic sclerosis and bronchiectasis was more prominent in younger participants than in older participants. This phenomenon could be explained by the fact that older participants have many factors that influence the development of bronchiectasis (i.e., antedated respiratory infections, tuberculosis history, and comorbid airway diseases), whereas bronchiectasis development in younger participants may be relatively more influenced by systemic sclerosis. Furthermore, if bronchiectasis occurs in younger individuals with systemic sclerosis, they would experience the development of respiratory symptoms and deterioration of the quality of life during treatment for systemic sclerosis. Because immunosuppressants are used for treating systemic sclerosis, patients with comorbid bronchiectasis may be prone to recurrent respiratory infections [35]. Thus, bronchiectasis might affect the prognosis in patients with systemic sclerosis so actively diagnosing and managing bronchiectasis would be of paramount importance [36].

Our study provides two relevant considerations for clinicians and researchers. First, an increased risk of bronchiectasis in individuals with systemic sclerosis suggests that bronchiectasis should be regarded as an important pulmonary comorbidity along with ILD and pulmonary hypertension. Early screening strategies to detect bronchiectasis might be helpful, especially in those at an early age, who are at a higher risk of developing bronchiectasis compared to controls. Second, the increased risk of bronchiectasis in systemic sclerosis warrants future studies on the relationship between the two diseases: studies focusing on whether bronchiectasis affects the natural course of systemic sclerosis. If so, management of bronchiectasis to modulate the treatment outcomes of systemic sclerosis is needed.

This study has some limitations. First, since this study was conducted in a Korean population, it may be difficult to generalize the study results to other countries and ethnic groups. Second, as the NHIS database does not contain specific data on smoking history, body mass index, lung function test, type of systemic sclerosis (limited vs. diffuse), related symptoms, and use of immunosuppressants, we could not include these factors in our analysis. Third, bronchiectasis was determined using ICD-10 codes. Thus, this study captured only bronchiectasis cases that were of sufficient clinical significance to lead clinicians to claim diagnostic codes for bronchiectasis, which implies an underestimation of the incidence estimates. Fourth, traction bronchiectasis associated with ILD might have been included as bronchiectasis in this study. However, sensitivity analysis evaluating the risk of bronchiectasis without a diagnosis code for ILD (ICD-10 code for bronchiectasis, but without ICD-10 code for ILD) showed similar results (Supplementary Tables 1–2 and Supplementary Fig. 1). Finally, our study had a relatively short follow-up period, which may have led to underestimation of the true incidence of bronchiectasis in patients with systemic sclerosis.

## Conclusions

A nationwide longitudinal database demonstrated that the risk of incident bronchiectasis is higher in individuals with systemic sclerosis than in those without. Because pulmonary complications play a major role in deciding mortality in patients with systemic sclerosis, clinicians need to identify and appropriately manage bronchiectasis in this population, particularly in younger people. Follow-up studies are needed to investigate how bronchiectasis in patients with systemic sclerosis affects the risk of infection, lung function, and quality of life.

### Supplementary Information

**Additional file 1: Supplemental Figure S1.** Cumulative incidence probability of bronchiectasis (/100,000 person-years) in systemic sclerosis and matched cohorts excluding ILD diagnosis codes. **Supplemental Table S1.** Baseline characteristics in systemic sclerosis and matched cohorts excluding ILD diagnosis codes. **Supplemental Table S2.** Risk of bronchiectasis according to the presence or absence of systemic sclerosis excluding ILD diagnosis codes. **Supplemental Table S3.** Subgroup analysis of the risk of bronchiectasis.

## Data Availability

All data extracted in this study are included in the current article.

## Abbreviations

ILD: Interstitial lung disease
NHIS: National Health Insurance Service
ICD: International Classification of Disease
CTD: Connective tissue disease
COPD: Chronic obstructive pulmonary disease
SD: Standard deviation
CI: Confidence interval
