# Supplementary material for: Systemic sclerosis and risk of bronchiectasis: a nationwide longitudinal cohort study
Source: Arthritis Res Ther. 2023 Oct 23;25:209. doi: 10.1186/s13075-023-03189-2 (PMC10591419; doi:10.1186/s13075-023-03189-2)
Supplement: Supplementary file 1 — Additional file 1: Supplemental Figure S1. Cumulative incidence probability of bronchiectasis (/100,000 person-years) in systemic sclerosis and matched cohorts excluding ILD diagnosis codes. Supplemental Table S1. Baseline characteristics in systemic sclerosis and matched cohorts excluding ILD diagnosis codes. Supplemental Table S2. Risk of bronchiectasis according to the presence or absence of systemic sclerosis excluding ILD diagnosis codes. Supplemental Table S3. Subgroup analysis of the risk of bronchiectasis. [file 13075_2023_3189_MOESM1_ESM.docx]

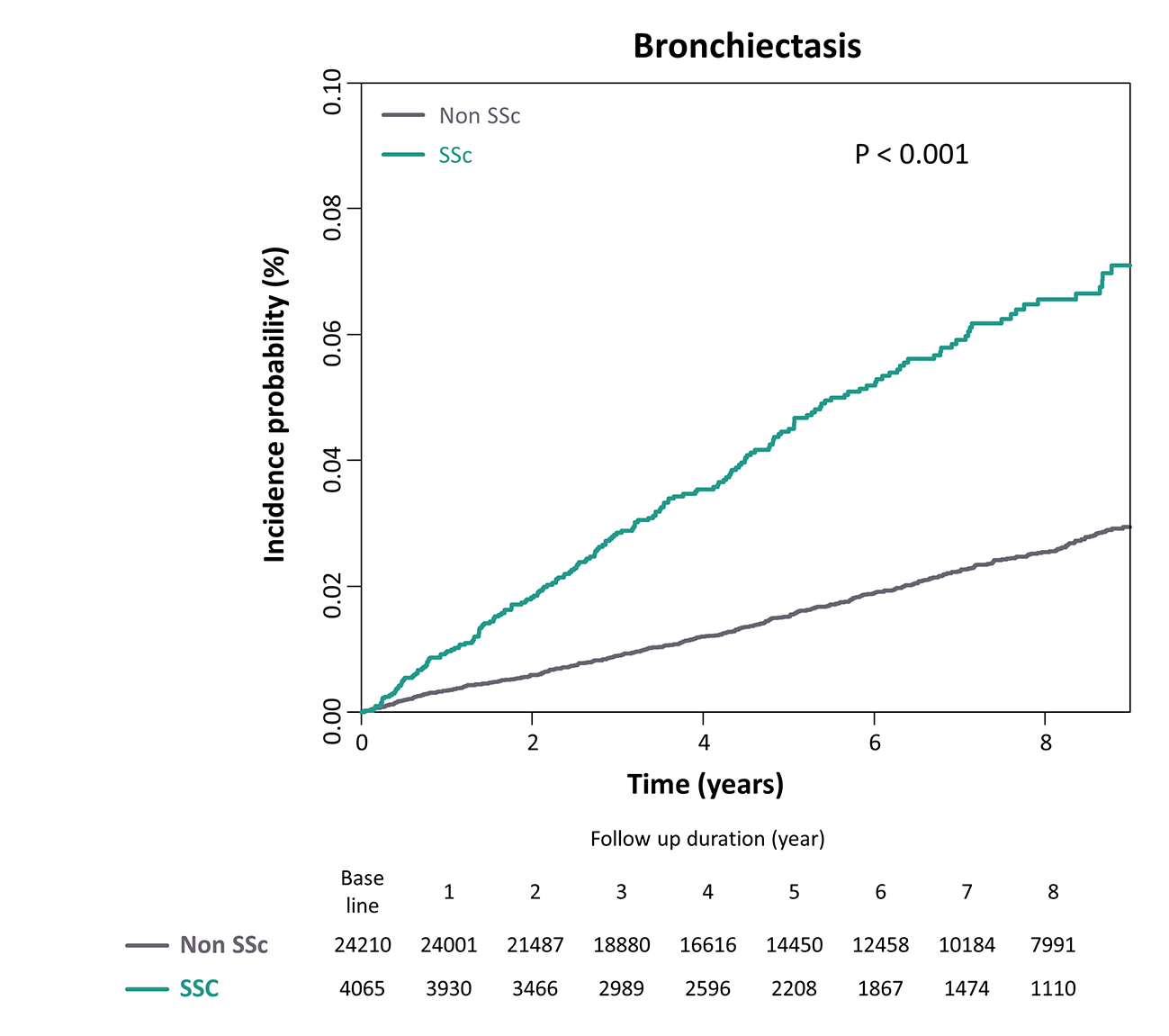


**Supplemental Figure S1.** Cumulative incidence probability of bronchiectasis (/100,000 person-years) in systemic sclerosis and matched cohorts excluding ILD diagnosis codes.

*Abbreviations*: ILD, interstitial lung disease.

**Supplemental Table S1.** Baseline characteristics in systemic sclerosis and matched cohorts excluding ILD diagnosis codes

|  | Total  (N = 14,154) | Systemic sclerosis cohort  (n = 2,359) | Matched cohort  (n = 11,795) | P value |
| --- | --- | --- | --- | --- |
| Male sex | 2,130 (15.1) | 355 (15.1) | 1,775 (15.1) | >0.999 |
| Age, years | 55.5 ± 10.7 | 55.5 ± 10.7 | 55.5 ± 10.7 | >0.999 |
| 20–39 | 858 (6.1) | 143 (6.1) | 715 (6.1) |  |
| 40–64 | 10,332 (73.0) | 1,722 (73.0) | 8,610 (73.0) |  |
| ≥65 | 2,964 (20.9) | 494 (20.9) | 2,470 (20.9) |  |
| Extra-pulmonary comorbidities |  |  |  |  |
| Diabetes mellitus | 1,585 (11.2) | 237 (10.1) | 1,348 (11.4) | 0.052 |
| Hypertension | 5,310 (37.5) | 1,349 (57.2) | 3,961 (33.6) | <0.001 |
| Dyslipidemia | 4,360 (30.8) | 677 (28.7) | 3,683 (31.2) | 0.015 |
| ESRD | 847 (5.9) | 151 (6.4) | 696 (5.9) | 0.350 |
| IHD | 1,011 (7.1) | 357 (15.1) | 654 (5.5) | <0.001 |
| CHF | 248 (1.8) | 106 (4.5) | 142 (1.2) | <0.001 |
| Pulmonary comorbidities |  |  |  |  |
| COPD | 1,307 (9.2) | 456 (19.3) | 851 (7.2) | <0.001 |
| Asthma | 1,748 (12.4) | 447 (18.9) | 1,301 (11.0) | <0.001 |
| Previous pulmonary tuberculosis | 30 (0.2) | 11 (0.5) | 19 (0.2) | <0.001 |
| CTD other than systemic sclerosis | 260 (1.8) | 209 (8.9) | 51 (0.4) | <0.001 |
| Rheumatoid arthritis | 104 (0.7) | 61 (2.6) | 43 (0.4) | <0.001 |
| Systemic lupus erythematous | 98 (0.7) | 91 (3.9) | 7 (0.1) | <0.001 |
| Dermatomyositis | 11 (0.1) | 11 (0.5) | 0 (0.0) | <0.001 |
| Mixed connective tissue disease | 61 (0.4) | 61 (2.6) | 0 (0.0) | <0.001 |
| Polymyalgia rheumatica | 2 (0.0) | 1 (0.0) | 1 (0.0) | 0.206 |

Data are presented as number (percentage) or mean ± standard deviation.

*Abbreviations:* ILD, interstitial lung disease; ESRD, end-stage renal disease; IHD, ischemic heart disease; CHF, congestive heart failure; COPD, chronic obstructive lung disease; CTD, connective tissue disease.

**Supplemental Table S2.** Risk of bronchiectasis according to the presence of systemic sclerosis excluding ILD diagnosis codes

|  |  |  |  |  | HR (95% CI) | | |
| --- | --- | --- | --- | --- | --- | --- | --- |
|  | N | Incident bronchiectasis (n) | Duration  (PY) | IR per 1,000 PY | Model 1 | Model 2 | Model 3 |
| Matched cohort | 11,795 | 222 | 60498.35 | 3.67 | 1 (reference) | 1 (reference) | 1 (reference) |
| Systemic sclerosis cohort | 2,359 | 122 | 11352.84 | 10.75 | 2.93 (2.35–3.66) | 3.02 (2.41–3.80) | 2.62 (2.06–3.33) |

Data are presented as a risk ratio (95% confidence interval).

Model 1 was an unadjusted model; Model 2 was adjusted for sex, age, income, diabetes mellitus, hypertension, and dyslipidemia; Model 3 was additionally adjusted for ESRD, IHD, CHF, COPD, asthma, tuberculosis, and CTD other than systemic sclerosis.

*CTD other than systemic sclerosis included rheumatoid arthritis, systemic lupus erythematosus, dermatomyositis, mixed connective tissue disease, and polymyalgia rheumatica.

*Abbreviations:* ILD, interstitial lung disease; HR, hazard ratio; CI, confidence interval; PY, person-years; IR, incidence rate; ESRD, end-stage renal disease; IHD, ischemic heart disease; CHF, congestive heart failure; COPD, chronic obstructive pulmonary disease; CTD, connective tissue disease.

**Supplemental Table S3.** Subgroup analysis of the risk of bronchiectasis in patients with systemic sclerosis

|  |  |  |  |  |  | **HR (95% CI)** | | |
| --- | --- | --- | --- | --- | --- | --- | --- | --- |
| **Subgroups** | **Systemic sclerosis** | **N** | **Incident bronchiectasis**  **(n)** | **Duration**  **(PY)** | **IR per 1,000 PY** | **Model 1** | **Model 2** | **Model 3** |
| *Other Comorbidities* |  |  |  |  |  |  |  |  |
| **Diabetes mellitus** |  |  |  |  |  |  |  |  |
| No | No | 22,259 | 407 | 130,556 | 3.12 | 1 (reference) | 1 (reference) | 1 (reference) |
|  | Yes | 4,456 | 241 | 24,080 | 10.01 | 3.21 (2.74–3.76) | 3.15 (2.6–3.72) | 2.68 (2.25–3.20) |
| Yes | No | 1,966 | 48 | 10,307 | 4.66 | 1 (reference) | 1 (reference) | 1 (reference) |
|  | Yes | 389 | 18 | 1,846 | 9.75 | 2.09 (1.22–3.60) | 2.412(1.40–4.15) | 2.13 (1.24–3.68) |
| *p* for interaction |  |  |  |  |  | 0.138 | 0.354 | 0.429 |
| **Hypertension** |  |  |  |  |  |  |  |  |
| No | No | 18,376 | 308 | 108,550 | 2.84 | 1 (reference) | 1 (reference) | 1 (reference) |
|  | Yes | 2,283 | 94 | 12,406 | 7.58 | 2.67 (2.12–3.36) | 2.69 (2.13–3.39) | 2.29 (1.80–2.91) |
| Yes | No | 5,849 | 147 | 32,313 | 4.55 | 1 (reference) | 1 (reference) | 1 (reference) |
|  | Yes | 2,562 | 165 | 13,519 | 12.20 | 2.68 (2.14–3.35) | 3.51 (2.80–4.41) | 3.01 (2.38–3.81) |
| *p* for interaction |  |  |  |  |  | 0.985 | 0.106 | 0.098 |
| **Dyslipidemia** |  |  |  |  |  |  |  |  |
| No | Yes | 20,311 | 356 | 121,058 | 2.94 | 1 (reference) | 1 (reference) | 1 (reference) |
|  | No | 3,923 | 219 | 21,524 | 10.17 | 3.46 (2.92–4.09) | 3.42 (2.86–4.07) | 2.89 (2.40–3.48) |
| Yes | Yes | 3,914 | 99 | 19,805 | 5.00 | 1 (reference) | 1 (reference) | 1 (reference) |
|  | No | 922 | 40 | 4,401 | 9.09 | 1.82 (1.26–2.63) | 2.00 (1.38–2.89) | 1.81 (1.25–2.62) |
| *p* for interaction |  |  |  |  |  | 0.001 | 0.010 | 0.024 |
| **IHD** |  |  |  |  |  |  |  |  |
| No | No | 22,954 | 425 | 133,884 | 3.17 | 1 (reference) | 1 (reference) | 1 (reference) |
|  | Yes | 4,125 | 213 | 22,400 | 9.51 | 2.99 (2.54–3.53) | 2.98 (2.52–3.54) | 2.56 (2.14–3.06) |
| Yes | No | 1,271 | 30 | 6,978 | 4.30 | 1 (reference) | 1 (reference) | 1 (reference) |
|  | Yes | 720 | 46 | 3,526 | 13.05 | 3.03 (1.91–4.80) | 3.80 (2.39–6.03) | 3.20 (2.01–5.11) |
| *p* for interaction |  |  |  |  |  | 0.962 | 0.337 | 0.370 |
| **CHF** |  |  |  |  |  |  |  |  |
| No | No | 23,917 | 450 | 139,475 | 3.23 | 1 (reference) | 1 (reference) | 1 (reference) |
|  | Yes | 4,614 | 244 | 25,124 | 9.71 | 3.01 (2.57–3.52) | 3.00 (2.55–3.53) | 2.57 (2.16–3.05) |
| Yes | No | 308 | 5 | 1,388 | 3.60 | 1 (reference) | 1 (reference) | 1 (reference) |
|  | Yes | 231 | 15 | 801 | 18.71 | 5.21 (1.89–14.35) | 6.79 (2.46–18.72) | 5.87 (2.12–16.20) |
| *p* for interaction |  |  |  |  |  | 0.293 | 0.118 | 0.114 |
| **ESRD** |  |  |  |  |  |  |  |  |
| No | No | 24,172 | 453 | 140,622 | 3.22 | 1 (reference) | 1 (reference) | 1 (reference) |
|  | Yes | 4,810 | 257 | 25,793 | 9.96 | 3.09 (2.65–3.60) | 3.08 (2.62–3.61) | 2.63 (2.22–3.12) |
| Yes | No | 53 | 2 | 241 | 8.30 | 1 (reference) | 1 (reference) | 1 (reference) |
|  | Yes | 35 | 2 | 133 | 15.04 | 1.82 (0.25–12.9) | 2.07 (0.29–14.7) | 1.78 (0.25–12.7) |
| *p* for interaction |  |  |  |  |  | 0.598 | 0.692 | 0.697 |

Data are presented as hazard ratios (95% confidence intervals).

Model 1 was an unadjusted model; Model 2 was adjusted for sex, age, income, diabetes mellitus, hypertension, and dyslipidemia; Model 3 was additionally adjusted for ESRD, IHD, CHF, COPD, asthma, tuberculosis, and CTD other than systemic sclerosis.

CTD other than systemic sclerosis included rheumatoid arthritis, systemic lupus erythematosus, dermatomyositis, mixed connective tissue disease, and polymyalgia rheumatica.

*Abbreviations:* HR, hazard ratio; CI, confidence interval; PY, person-years; IR, incidence rate; ESRD, end-stage renal disease; IHD, ischemic heart disease; CHF, congestive heart failure; COPD, chronic obstructive pulmonary disease; CTD, connective tissue disease.
